# Development of Wide-Compatible *Indica* Lines by Pyramiding Multiple Neutral Alleles of *Indica*–*Japonica* Hybrid Sterility Loci

**DOI:** 10.3389/fpls.2022.890568

**Published:** 2022-04-29

**Authors:** Jie Guo, Yun Li, Liang Xiong, Tingxian Yan, Jinsong Zou, Ziju Dai, Guang Tang, Kangli Sun, Xin Luan, Weifeng Yang, Quanya Tan, Haitao Zhu, Ruizhen Zeng, Shaokui Wang, Guiquan Zhang

**Affiliations:** ^1^Guangdong Provincial Key Laboratory of Plant Molecular Breeding, State Key Laboratory for Conservation and Utilization of Subtropical Agro-Bioresources, South China Agricultural University, Guangzhou, China; ^2^Rice Research Institute, Guangdong Academy of Agricultural Sciences, Guangzhou, China

**Keywords:** hybrid rice, heterosis, hybrid sterility, neutral allele, breeding by design

## Abstract

Since the development of *indica* hybrid rice in the 1970s, great success has been achieved in hybrid rice production in China and around the world. The utilization of inter-subspecific *indica–japonica* hybrid rice has always been considered due to its stronger heterosis characteristics. However, *indica*–*japonica* hybrids face a serious problem of sterility, which hinders the exploitation of their heterosis. In the past decades, the genetic basis of *indica*–*japonica* hybrid sterility has been well studied. It was found that in sterile *indica*–*japonica* hybrids, female sterility was mainly controlled by the *S5* locus and male sterility by the *Sa*, *Sb*, *Sc*, *Sd*, and *Se* loci. In this study, we developed wide-compatible *indica* lines (WCILs) by pyramiding multiple neutral (n) alleles of the hybrid sterility loci. First, we identified *S^n^* alleles of the loci in single-segment substitution lines (SSSLs) in the genetic background of *indica* Huajingxian 74 (HJX74). Then, the *S^n^* alleles of *S5*, *Sb*, *Sc*, *Sd*, and *Se* loci in SSSLs were pyramided in the HJX74 genetic background. The WCILs carrying *S^n^* alleles at the *S5*, *Sb*, *Sc*, *Sd*, and *Se* loci showed wide compatibility with *indica* and *japonica* rice varieties. Therefore, the WCILs will be used to develop inter-subspecific *indica–japonica* hybrid rice with normal fertility.

## Introduction

Asian cultivated rice (*Oryza sativa* L.) is the staple food for more than half of the world’s population (Fukagawa and Ziska, 2019). The breeding of high-yielding varieties is essential for maintaining global food security (Peng et al., 2008; Khush, 2013). Since the 1970s, *indica* hybrid rice has been successfully developed in China and around the world (Yuan and Virmani, 1988; Cheng et al., 2007). However, the heterosis of intra-subspecific hybrid rice is limited, resulting in a yield plateau for production of hybrid rice (Peng et al., 2004; Cheng et al., 2007). There is great heterosis in inter-subspecific hybrids, and exploiting this heterosis has long been considered a promising approach to further increase the yield potential of rice (Khush, 2013; Zhang et al., 2021). However, the severe sterility associated with *indica*–*japonica* hybrid hinders the utilization of heterosis (Ikehashi and Araki, 1986; Ouyang and Zhang, 2018; Zhang, 2020, 2022).

The sterility of hybrids produced by crossing *indica* and *japonica* rice varieties can be attributed to female or embryo sac sterility and male or pollen sterility. The female sterility in hybrid is mainly controlled by the *S5* locus, which was mapped on chromosome 6 (Ikehashi and Araki, 1986; Yanagihara et al., 1995; Ji et al., 2005; Qiu et al., 2005). The male sterility in hybrid is mainly controlled by *Sa*, *Sb*, *Sc*, *Sd*, and *Se* loci (Zhang and Lu, 1989, 1993; Zhang et al., 1993, 1994). Using molecular markers, *Sa* was found to be located on chromosome 1 (Zhuang et al., 1999; Su and Liu, 2003), *Sb* on chromosome 5 (Zhuang et al., 2002; Li et al., 2006), *Sc* on chromosome 3 (Zhang and Zhang, 2001; Yang et al., 2004), *Sd* on chromosome 1 (Li et al., 2008), and *Se* on chromosome 12 (Zhu et al., 2008). The *S5*, *Sa*, and *Sc* genes were then cloned and functionally analyzed (Chen et al., 2008; Long et al., 2008; Yang et al., 2012; Shen et al., 2017). The genetic model of hybrid sterility is the one-locus sporo-gametophytic interaction model (Ikehashi and Araki, 1986; Zhang and Lu, 1993; Zhang, 2020). In this genetic model, it is assumed that *indica* varieties have *S^i^* allele, and *japonica* varieties have *S^j^* allele at the loci. At the *S5* locus, the interaction between *S^i^* and *S^j^* causes the abortion of female gametes carrying *S^j^* allele (Ikehashi and Araki, 1986). At the *Sa*, *Sb*, *Sc*, *Sd*, and *Se* loci, the interaction between *S^i^* and *S^j^* causes the abortion of male gametes carrying *S^j^* allele (Zhang and Lu, 1993). At these loci, some varieties carry *S^n^*, a neutral allele, and the allelic interaction between *S^i^*/*S^n^* and *S^j^*/*S^n^* cannot cause the abortion of any gamete (Ikehashi and Araki, 1986; Zhang and Lu, 1993; Yang et al., 2012; Shen et al., 2017; Xie et al., 2017). The understanding of the genetic and molecular mechanisms of sterility in *indica–japonica* hybrids has laid the foundation for overcoming hybrid sterility.

With the development of molecular breeding technology, the concept of “breeding by design” was proposed (Peleman and van der Voort, 2003). To implement the strategy of rice breeding by design, a library of single-segment substitution lines (SSSLs) in rice was constructed by using 43 accessions from seven species of AA genome as donors of chromosome substitution segments in the genetic background of Huajingxian 74 (HJX74), an elite *indica* variety from south China. A total of 2,360 HJX74-SSSLs have been included in the library, which contains rich genetic resources for rice breeding techniques (Zhang et al., 2004; Xi et al., 2006; He et al., 2017; Zhao et al., 2019; Zhang, 2021). The HJX74-SSSL library was used as a platform for designing new rice cultivars, and several cytoplasmic male sterility (CMS), maintainer, and restorer lines were developed (Dai et al., 2015, 2016; Luan et al., 2019). Therefore, target chromosome-segment substitution is a way to breeding by design in rice (Zhang, 2021).

With the understanding of the genetic and molecular mechanisms of *indica–japonica* hybrid sterility and the development of molecular breeding techniques, the breeding strategies for developing inter-subspecific *indica–japonica* hybrid rice were proposed (Zhang, 2020, 2022). One strategy for overcoming the hybrid sterility of *indica–japonica* rice is to develop *indica*-compatible *japonica* lines (ICJLs) (Zhang and Lu, 1999; Zhang, 2020). Recently, the ICJLs were developed by pyramiding *S^i^* allele at the *Sb*, *Sc*, *Sd*, and *Se* loci and *S^n^* allele at the *S5* locus in *japonica* genetic background by marker-assisted selection (MAS). The ICJLs are compatible with *indica* but incompatible with *japonica* in pollen fertility and spikelet fertility (Guo et al., 2016). Another strategy for overcoming the hybrid sterility of *indica–japonica* rice is to develop wide-compatible *indica* lines (WCILs) (Zhang, 2020, 2022). Herein, we report the development of WCILs using the HJX74-SSSL library. By pyramiding *S^n^* allele at the *S5*, *Sb*, *Sc*, *Sd*, and *Se* loci in the HJX74 genetic background, the obtained WCILs were compatible with both *indica* and *japonica* rice in pollen fertility and spikelet fertility. The breeding of WCILs provides a technique to develop inter-subspecific *indica–japonica* hybrid rice.

## Materials and Methods

### Plant Materials and Field Trials

Seven SSSLs carrying the *Sc* gene for hybrid male sterility in their chromosome substitution segments and seven SSSLs carrying the *S5* gene for hybrid female sterility in their chromosome substitution segments were selected from the HXJ74-SSSL library (Supplementary Table 1). A set of *indica* and *japonica* varieties were used as testers to test the hybrid fertility. The genotypes of *Sa*, *Sb*, *Sc*, *Sd*, and *Se* loci for hybrid male sterility and *S5* locus for hybrid female sterility have been identified in some of the testers. It was found that at these six loci, the *indica* variety Guang-lu-ai 4 (GLA4) carried the *S^i^* alleles, while the *japonica* variety Taichung 65 (T65) carried the *S^j^* alleles (Zhang et al., 1994; Guo et al., 2016). All the study samples were planted from 2008 to 2019 at the farm of South China Agricultural University, Guangzhou (23°07′N, 113°15′E). These plants were planted in two cropping seasons each year, with the first cropping season (FCS) running from late February to mid-July and the second cropping season (SCS) running from late July to mid-November. Seeds were sown in seedbeds, and seedlings were transplanted into the field. Field management, including irrigation, fertilization, and pest control, followed normal agricultural practices.

### Genotyping by Molecular Markers

The SSR markers were selected on the rice microsatellite maps (McCouch et al., 2002; Zhang et al., 2007). The functional markers of the *S5* gene were selected to identify the genotypes at the *S5* loci (Sundaram et al., 2010; Du et al., 2011; Yang et al., 2012; Guo et al., 2016). Markers linked with the *Sa, Sb, Sc, Sd, Se*, and *S5* loci were selected from the published studies (Yang et al., 2004, 2012; Li et al., 2006, 2008; Chen et al., 2008; Long et al., 2008; Zhu et al., 2008). New molecular markers were developed in this study (Supplementary Table 2). The PCR products were separated into 6% non-denaturing polyacrylamide gels (Panaud et al., 1996; Li et al., 2006).

### Phenotyping of Fertility and Agronomic Traits

To check pollen fertility, nine mature flowers were collected from the upper third of panicles during the flowering stage and fixed in FAA solution. Pollens were stained with the 1% I_2_-KI solution containing 0.1% (w/v) iodine and 1% (w/v) potassium iodide. Pollens were divided into normal pollens and sterile pollens, which were further divided into stained abortive pollens (stained but small size) and empty abortive pollens (small size and empty) (Zhang and Lu, 1989). Three panicles per plant and 10–12 plants per line were used to examine the spikelet fertility, and 20–40 plants per line were used to investigate the agronomic traits.

### Statistical Analysis

For statistical analysis, the percentage data were converted to the square root of the arcsine values. Student’s *t*-test was used to compare the data between the two groups. The Dunnett *t*-test was used to compare multiple groups with the control group. The least significance range (LSR) was used for the multiple range test among the multiple groups. The chi-square (χ^2^) test was performed to detect the distorted segregation of three genotypes in F_2_ populations according to the Mendelian ratio of 1:2:1. SPSS statistics 23.0 and Origin Pro 9.0 were used for data analysis and charting^1^.

## Results

### Genotypes of *Sa*, *Sb*, *Sc*, *Sd*, *Se*, and *S5* Loci in Huajingxian 74

**FIGURE 1:**
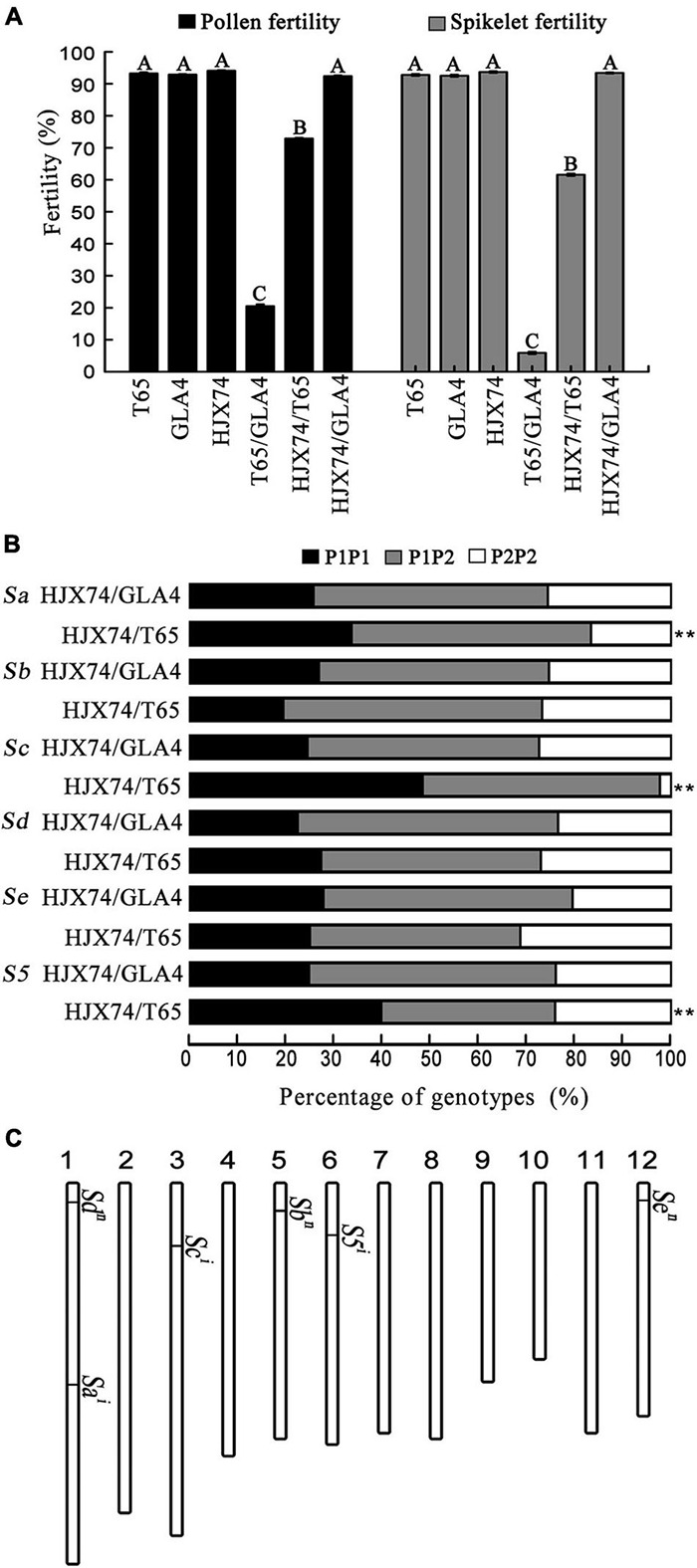
Genotypes of hybrid sterility loci *Sa*, *Sb*, *Sc*, *Sd*, *Se*, and *S5* in HJX74. **(A)** Pollen and spikelet fertility of three parents and their F_1_ hybrids. **(B)** Ratios of genotypes at *Sa*, *Sb*, *Sc*, *Sd*, *Se*, and *S5* loci in the F_2_ populations from the crosses between HJX74 and testers Taichung 65 (T65) and Guang-lu-ai 4 (GLA4). **(C)** Chromosome location and genotypes of the genes at the *Sa*, *Sb*, *Sc*, *Sd*, *Se*, and *S5* loci in HJX74. Vertical bars represent rice chromosomes. P1P1, Genotypes of HJX74; P1P2, Heterozygous genotype; P2P2, Genotype of testers T65 (*S^j^*/*S^j^*) or GLA4 (*S^i^*/*S^i^*). Capital letters indicate statistical differences at the 0.01 probability level.

To identify the genotypes of *Sa*, *Sb*, *Sc*, *Sd*, *Se*, and *S5* loci associated with hybrid sterility, HJX74 was test crossed with T65, a *japonica* variety with *S^j^* alleles at these six loci, and GLA4, an *indica* variety with *S^i^* alleles at these six loci (Zhang et al., 1994; Guo et al., 2016). The F_1_ hybrids obtained from the cross of T65/GLA4 showed severe sterility, where the pollen fertility was only 20.51% and spikelet fertility was only 5.89%. In contrast, the F_1_ hybrid of the HJX74/GLA4 cross showed normal pollen fertility and spikelet fertility of 92.39% and 93.39%, respectively. In the F_1_ hybrids obtained from the cross of HJX74/T65, the pollen fertility was 72.89% and the spikelet fertility was 61.58%, which were significantly higher than those of T65/GLA4 and significantly lower than those of HJX74/GLA4 hybrids (Figure 1A). The results showed that the hybrid of HJX74/T65 exhibited partial pollen sterility and partial spikelet sterility.

The molecular markers linked to the *Sa*, *Sb*, *Sc*, *Sd*, *Se*, and *S5* loci were used to investigate genotype segregation in the F_2_ populations obtained from the crosses of HJX74/GLA4 and HJX74/T65. At the *Sb*, *Sd*, and *Se* loci, the genotype segregation of F_2_ populations from both crosses fit the Mendelian ratio of 1:2:1. At the *Sa*, *Sc*, and *S5* loci, distorted segregation of the genotypes was detected in the F_2_ population of HJX74/T65 but not in the genotypes of HJX74/GLA4. At the *Sa* locus, the genotype ratios of *Sa^*HJX*74^*/*Sa^*HJX*74^*, *Sa^*HJX*74^*/*Sa^*T*65^*, and *Sa^*T*65^*/*Sa^*T*65^* were 68:100:33, which significantly distorted from the Mendelian ratio of 1:2:1. At the *Sc* locus, the genotype ratios of *Sc^*HJX*74^*/*Sc^*HJX*74^*, *Sc^*HJX*74^*/*Sc^*T*65^*, and *Sc^*T*65^*/*Sc^*T*65^* were 69:70:3, which significantly distorted from the Mendelian ratio. Distorted segregation was also detected at the *S5* locus, where the genotype ratios of *S5^*HJX*74^*/*S5^*HJX*74^*, *S5^*HJX*74^*/*S5^*T*65^*, and *S5^*T*65^*/*S5^*T*65^* were found to be 72:65:43 (Figure 1B). In addition, HJX74 was tested using a group of *indica* and *japonica* testers. The results showed that distorted segregation was detected only at the *Sa*, *Sc*, and *S5* loci in the crosses of HJX74/*japonica* testers (Supplementary Table 3).

These results indicated that HJX74 carried *S^i^*/*S^i^* at the *Sa*, *Sc*, and *S5* loci and *S^n^*/*S^n^* at the *Sb*, *Sd*, and *Se* loci (Figure 1C). At the *Sa* and *Sc* loci, the allele interaction between *S^i^* of HJX74 and *S^j^* of *japonica* testers caused the abortion of male gametes carrying *S^j^* in hybrids, resulting in the significant reduction of plants with *S^j^*/*S^j^* in the F_2_ populations. At the *S5* locus, the allele interaction between *S5^i^* of HJX74 and *S5^j^* of *japonica* testers caused the abortion of female gametes carrying *S5^j^* in hybrids, resulting in the significant reduction of plants with *S5^j^*/*S5^j^* in the F_2_ populations. At the *Sb*, *Sd*, and *Se* loci, allele interaction between *S^n^* of HJX74 and *S^j^* of *japonica* testers or *S^i^* of *indica* testers could not cause the abortion of any gamete in hybrids, and genotype segregation in the F_2_ populations fit the Mendelian ratio of 1:2:1 (Supplementary Table 3). In addition, compared with the *Sc* locus, the *Sa* locus showed weak distorted segregation, where χ^2^_(1:2:1)_ = 34.00–62.27 in the five segregation populations of the *Sc* locus, while χ^2^_(1:2:1)_ = 9.36–12.19 in the three segregation populations of the *Sa* locus (Supplementary Table 3). The results showed that the hybrid male sterility caused by the interaction between *S^i^* and *S^j^* at the *Sa* locus was weaker than that at the *Sc* locus.

### Genotypes of the *Sc* Locus in the Substitution Segments of Single-Segment Substitution Lines

**FIGURE 2:**
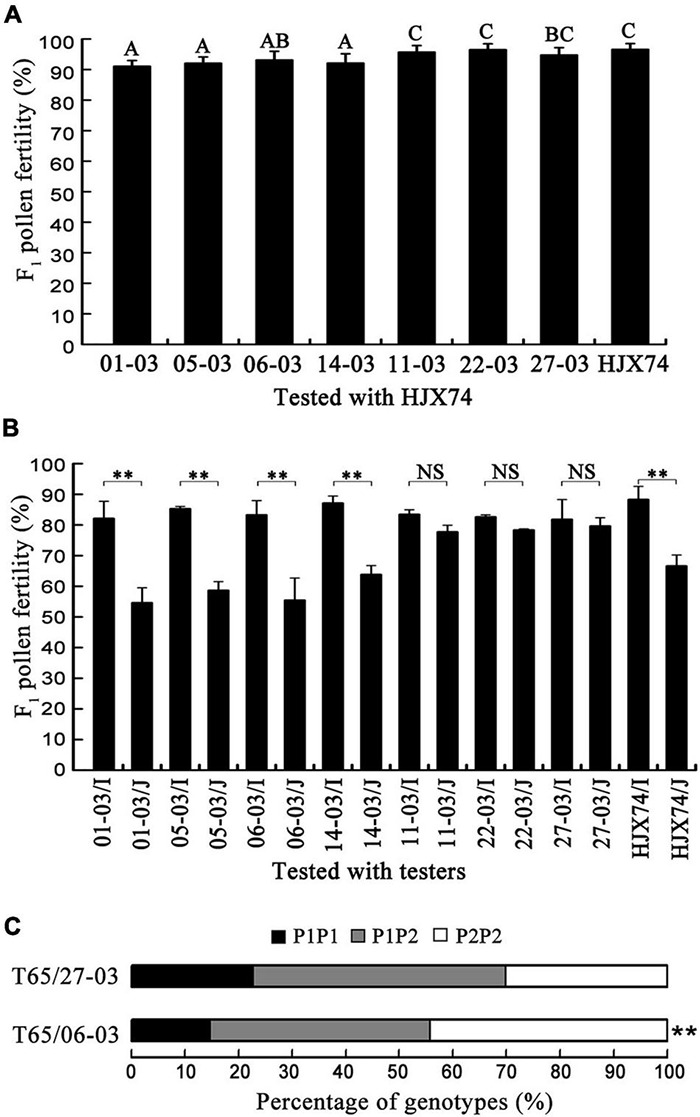
Genotypes and genetic effects of the *Sc* gene on chromosomal substitution segments of SSSLs with the HJX74 genetic background. **(A)** Pollen fertility of F_1_ hybrids from crosses between SSSLs and HJX74. **(B)** Pollen fertility of F_1_ hybrids from crosses between SSSLs and testers. I, *indica* testers; J, *japonica* testers. **(C)** Segregation ratios of *Sc* genotypes in F_2_ populations from crosses between SSSLs and T65. P1P1, Genotype of T65 (*S^j^*/*S^j^*); P1P2, Heterozygous genotypes; P2P2, Genotypes of SSSLs. ^**^, Significant difference at 0.01 probability level. NS, No significance.

To screen the *Sc^n^* gene, seven SSSLs carrying the *Sc* locus on the substitution segments obtained from different donors were selected from the HJX74-SSSL library (Supplementary Table 1). The pollen fertility of F_1_ hybrids from the crosses between the SSSLs and HJX74 was over 90% (Figure 2A). The SSSLs were then tested with three *indica* testers and three *japonica* testers. Four SSSLs (01-03, 05-03, 06-03, and 14-03) and HJX74 showed significantly higher pollen fertility in their F_1_ hybrids with *indica* testers than those obtained with *japonica* testers. In contrast, the other three SSSLs (11-03, 22-03, and 27-03) did not show a significant difference in the pollen fertility of F_1_ hybrids between the crosses with *indica* and *japonica* testers (Figure 2B). Two SSSLs (06-03 and 27-03) were then selected to detect the segregation of *Sc* genotypes in F_2_ populations obtained from the crosses with T65. In the F_2_ population of the T65/27-03 cross, the *Sc* genotypes of T65/T65, T65/27-03, and 27-03/27-03 segregated in the ratios of 40:83:53, which fit the Mendelian ratio of 1:2:1. In contrast, in the F_2_ population of the T65/06-03 cross, the genotype ratios of T65/T65, T65/06-03, and 06-03/06-03 were 23:64:69, which significantly distorted from the Mendelian ratio (Figure 2C). These results indicated that at the *Sc* locus, SSSLs 11-03, 22-03, and 27-03 carried the *S^n^* allele, while 01-03, 05-03, 06-03, and 14-03 carried the *S^i^* allele.

### Genotypes of the *S5* Locus in the Substitution Segments of Single-Segment Substitution Lines

To screen the *S5^n^* gene, seven SSSLs carrying the *S5* locus in the substitution segments obtained from different donors were selected from the HJX74-SSSL library (Supplementary Table 1). The genotypes of the *S5* locus in the SSSLs were detected by functional markers. The results showed that in the substitution segments, three SSSLs (04-06, 13-06, and 14-06) carried *S5^*i*^*, one SSSL (10-06) carried *S5^j^*, and the other three SSSLs (21-06, 23-06, and 27-06) carried *S5^n^* (Supplementary Table 4).

**FIGURE 3:**
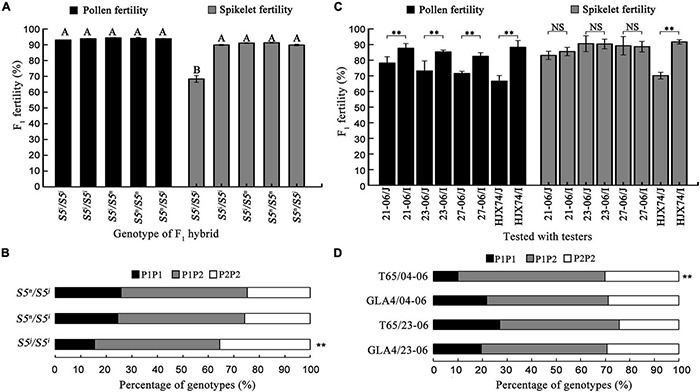
Genotypes and genetic effects of the *S5* gene on chromosomal substitution segments of SSSLs with the HJX74 genetic background. **(A)** Pollen fertility and spikelet fertility in F_1_ hybrids from the crosses between SSSLs and different *S5* genotypes. **(B)** Segregation ratios of *S5* genotypes in F_2_ populations from different crosses. **(C)** Pollen fertility and spikelet fertility in F_1_ hybrids from crosses between SSSLs (21-06, 23-06, and 27-06) and testers. I, *indica* testers; J, *japonica* testers. Capital letters indicate statistical differences at the 0.01 probability level. **(D)** Segregation ratios of *S5* genotypes in F_2_ populations from crosses between SSSLs and testers. P1P1, Genotype of tester; P1P2, Heterozygous genotypes; P2P2, Genotypes of SSSLs. ^**^, Significant difference at 0.01 probability level. NS, No significance.

Five genotypes of the *S5* locus were obtained from the F_1_ hybrids crossed by seven SSSLs (Supplementary Table 5). The pollen fertility of hybrids was normal in all crosses, ranging from 93.04 to 94.42%. The spikelet fertility of *S5^*i*^/S5^*i*^*, *S5^*i*^/S5^*n*^*, *S5^*n*^/S5^*j*^*, and *S5^*n*^/S5^*n*^* genotypes was normal (from 89.84% to 91.28%), but that of *S5^*i*^/S5^*j*^* genotype from the crosses between 10-06 carrying *S5^*j*^/S5^*j*^* and SSSLs carrying *S5^*i*^/S5^*i*^* was only 68.34%, which was significantly lower than the spikelet fertility of the other four genotypes (Figure 3A and Supplementary Table 5). The segregation of *S5* genotypes in F_2_ populations obtained from three heterozygous genotypes, *S5^*n*^/S5^*j*^, S5^*n*^/S5^*i*^*, and *S5^*j*^/S5^*i*^*, was detected by using the functional markers of the *S5* gene. Distorted segregation was detected in the *S5^*j*^/S5^*i*^* segregation population produced from the crosses between 10-06 carrying *S5^*j*^/S5^*j*^* and SSSLs carrying *S5^*i*^/S5^*i*^*, but was not detected in the segregation populations of *S5^*n*^/S5^*j*^* from 21-06/10-06 and of *S5^*n*^/S5^*i*^* from 21-06/13-06 (Figure 3B).

The three SSSLs with *S5^n^*, 21-06, 23-06, and 27-06, were tested for their wide compatibility by crossing with *indica* and *japonica* testers. The F_1_ hybrids from all crosses showed high spikelet fertility, from 80.28% to 95.05%. As a control, the spikelet fertility of F_1_ hybrids in HJX74/*japonica* testers was 70.13% (Figure 3C). In addition, distorted segregation of the *S5* locus was detected in the F_2_ population of T65/04-06, but was not detected in the F_2_ populations of the other three crosses, that is, GLA4/04-06, T65/23-06, and GLA4/23-06 (Figure 3D). These results showed that the three SSSLs (21-06, 23-06, and 27-06) were compatible with *indica* testers and *japonica* testers in spikelet fertility as a result of their carrying *S5^n^* locus.

### Pyramiding of *S^n^* Alleles at the *Sc* and *S5* Loci in the Huajingxian 74 Genetic Background

**FIGURE 4:**
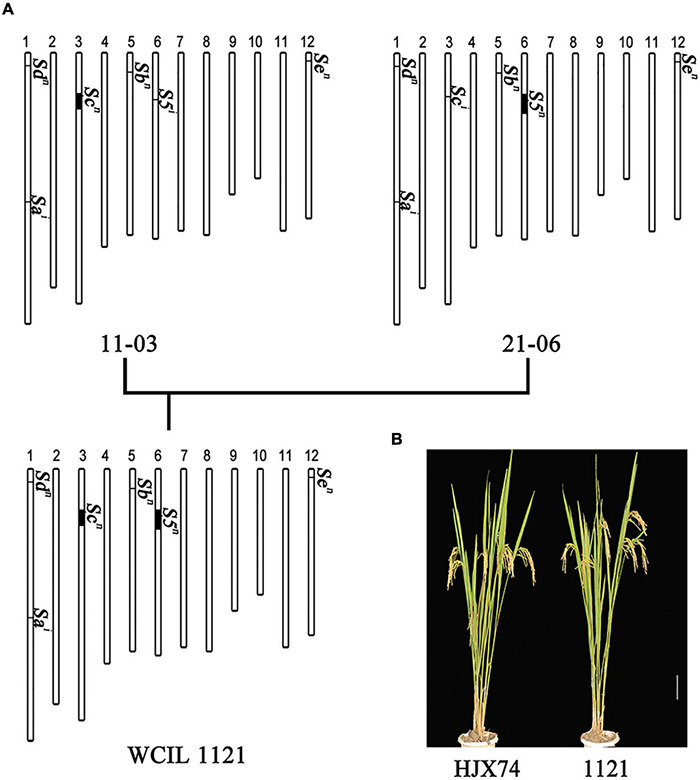
Pyramiding of *S^n^* genes at the *Sc* and *S5* loci in the HJX74 genetic background. **(A)** Development of WCIL 1121 by pyramiding of *Sc^n^* in the substitution segment of SSSL 11-03 and *S5^n^* in the substitution segment of SSSL 21-06. Scale bar, 2 cm. Vertical bars represent rice chromosomes. Deep parts represent the substitution segments from donors and light parts represent the genetic background of HJX74. **(B)** Plant types of WCIL 1121 and HJX74. Scale bar, 10 cm.

Three SSSLs (11-03, 22-03, and 27-03) with the *Sc^n^* gene and three SSSLs (21-06, 23-06, and 27-06) with the *S5^n^* gene were selected to pyramid the two *S^n^* genes in the HJX74 genetic background. Three SSSLs with *Sc^n^* were crossed with three SSSLs with *S5^n^*, respectively. In the segregating populations, the plants carrying *Sc^n^* and *S5^n^* loci were selected. Nine pyramiding lines were developed, which carried *Sc^n^* and *S5^n^* loci from different donors and *Sb^n^*, *Sd^n^*, and *Se^n^* in the HJX74 genetic background (Figure 4A and Supplementary Table 6). Therefore, the nine pyramiding lines thus obtained were WCILs.

In the nine WCILs, the plant type was similar to HJX74 (Figure 4B). In addition, no significant difference between HJX74 and WCILs was found in the majority of the investigated traits, including heading date, plant height, width of flag leaf, length of flag leaf, grain length, grain width, and grain weight (Supplementary Table 7).

### Compatibility of Wide-Compatible *indica* Lines

**FIGURE 5:**
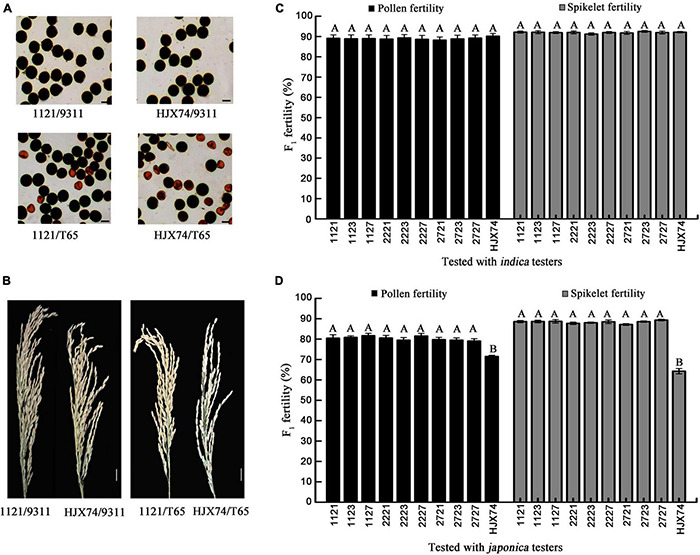
Compatibility of WCILs. **(A)** Pollen grains stained by I_2_-KI solution in F_1_ hybrids of four crosses. Scale bar, 30 μm. **(B)** Spikelet fertility of the panicles in F_1_ hybrids. Scale bar, 2 cm. **(C)** Pollen fertility and spikelet fertility of the F_1_ hybrids from the crosses between WCILs (HJX74 as control) and *indica* testers (See Supplementary Tables 8–9). **(D)** Pollen fertility and spikelet fertility of the F_1_ hybrids from the crosses between WCILs (HJX74 as control) and *japonica* testers (See Supplementary Tables 10–11). The information about nine WCILs (1121, 1123, 1127, 2221, 2223, 2227, 2721, 2723, and 2727) is given in Supplementary Table 6. HJX74 is a recipient of SSSLs. The 9311 is an *indica* tester. T65 is a *japonica* tester. Capital letters indicate statistical differences at the 0.01 probability level.

To evaluate the compatibility of nine WCILs, the WCILs were test crossed with six *indica* testers and five *japonica* testers (Supplementary Tables 8–11). When tested with *indica* tester group, F_1_ hybrids of nine WCILs showed normal pollen fertility and spikelet fertility, with no significant difference when compared to HJX74. When tested with the *japonica* tester group, nine WCILs showed significantly higher F_1_ pollen fertility and spikelet fertility when compared to HJX74 (Figure 5). These results indicated that the WCILs showed wide compatibility, producing high pollen fertility and spikelet fertility in their F_1_ hybrids with both *indica* and *japonica* rice varieties.

Although the WCILs showed significantly higher F_1_ pollen fertility when tested with *japonica* testers, the F_1_ pollen fertility was still lower when tested with *indica* testers (Figures 5C,D). To identify the problem, the genotype segregation at the *Sa, Sb, Sc, Sd, Se*, and *S5* loci in F_2_ populations was examined with molecular markers linked with these loci. No distorted segregation at the *Sb*, *Sc*, *Sd*, *Se*, and *S5* loci was found in all the detected F_2_ populations, further confirming the fact that the alleles of the *Sb*, *Sc*, *Sd*, *Se*, and *S5* loci in WCILs were *S^n^*. However, significantly distorted segregation was detected at the *Sa* locus in all four populations (Supplementary Table 12). These results verified that WCILs carried the *Sa^i^* gene in the HJX74 genetic background, and the interaction between *Sa^i^* from WCILs and *Sa^j^* from *japonica* testers caused some male gametes with *Sa^j^* to become abortive in F_1_ hybrids obtained from the crosses of WCILs with *japonica* testers.

Three *indica* lines, GLA4 carrying the genotype of *Sa^i^*, *Sb^i^*, *Sc^i^*, *Sd^i^*, *Se^i^*, and *S5^i^*, HJX74 carrying the genotype of *Sa^i^*, *Sb^n^*, *Sc^i^*, *Sd^n^*, *Se^n^*, and *S5^i^*, and WCIL 2223 carrying the genotype of *Sa^i^*, *Sb^n^*, *Sc^n^*, *Sd^n^*, *Se^n^*, and *S5^n^*, were selected to test their compatibility with eight *japonica* varieties of different ecotypes. In the F_1_ hybrids of GLA4 with eight *japonica* varieties, pollen fertility was 13.24–90.68% with an average of 46.94%, and spikelet fertility was 5.89–92.95% with an average of 44.40%. In the F_1_ hybrids of HJX74 with eight *japonica* varieties, pollen fertility was 75.19–95.74% with an average of 85.37%, and spikelet fertility was 58.34–93.90% with an average of 74.69%. On comparison of data, pollen fertility was 82.35–95.79% with an average of 88.82%, and spikelet fertility was 89.19–94.29% with an average of 91.73% in the F_1_ hybrids of WCIL 2223 with the eight *japonica* varieties (Supplementary Table 13). The results showed that WCIL 2223 had higher and wider compatibility with *japonica* varieties than GLA4 and HJX74. The pollen fertility and spikelet fertility in F_1_ hybrids of WCIL with various *japonica* varieties were normal or near normal.

## Discussion

### Sterility or Compatibility of Hybrids Between *indica* and *japonica* Subspecies Is a Complex Trait

In the past decades, the genetic basis of *indica*–*japonica* hybrid sterility has been understood. In *indica*–*japonica* hybrid sterility, the *S5* locus was found to be responsible for female sterility, and the *Sa*, *Sb*, *Sc*, *Sd*, and *Se* loci were responsible for male sterility. Following the tri-allele pattern and the one-locus sporo-gametophytic interaction model, the allele interaction between *S^i^* and *S^j^* leads to the abortion of male or female gametes carrying *S^j^*, whereas the allele interaction between *S^n^* and *S^i^* or *S^j^* does not lead to the abortion of any gamete (Zhang, 2020, 2022). Thus, the sterility or compatibility of hybrids between *indica* and *japonica* subspecies is a complex trait that is controlled by multiple genes. Due to the diversity of *indica* and *japonica* rice varieties, the genotypes of hybrid sterility vary greatly among different varieties, particularly modern varieties, resulting in different crossing combinations with different degrees of hybrid sterility. In addition, the effects of alleles obtained from different donors are quantitatively different, resulting in the continuous variation of fertility at a single locus (Zhang et al., 1993, 1994). The molecular basis of allele diversity has been revealed by the cloned genes of *S5* (Chen et al., 2008; Yang et al., 2012), *Sa* (Long et al., 2008; Xie et al., 2017), and *Sc* (Shen et al., 2017). In this study, we found that HJX74, the recipient of SSSLs, carried the *S^n^* allele at the *Sb*, *Sd*, and *Se* loci but the *S^i^* allele at the *S5*, *Sa*, and *Sc* loci (Figure 1). In addition, the effect of *Sa^i^* was weaker than that of *Sc^i^* in HJX74 (Supplementary Table 3). The identification of genotypes that lead to hybrid sterility provided a prerequisite for improving the compatibility of HJX74.

### Hybrid Sterility in *indica–japonica* Rice Can Be Overcome by Developing ICJLs and Wide-Compatible *indica* Lines

Based on the tri-allele pattern and the one-locus sporo-gametophytic interaction model, the *indica*–*japonica* hybrid sterility can be overcome by developing ICJLs and WCILs (Zhang and Lu, 1999; Zhang, 2020, 2022). ICJLs can be developed by transferring the *S^i^* allele from *indica* to *japonica* rice. In hybrids between *indica* varieties having *S^i^* allele and ICJLs having *S^i^* allele in *japonica* genetic background, the *S^*i*^/S^*i*^* genotype cannot cause the abortion of any gamete. In a previous study, we transferred the *S^i^* allele from *indica* donors to the *japonica* T65 variety to develop ICJLs, which carry the *S^i^* allele at hybrid sterility loci in the *japonica* genetic background. The result was that ICJLs were compatible with *indica* but incompatible with *japonica* rice (Guo et al., 2016). In another method, WCILs can be developed by transferring the *S^n^* allele from donors to *indica* rice. In hybrids between WCILs having *S^n^* allele in *indica* genetic background and *japonica* varieties having *S^j^* allele, the *S^*n*^/S^*j*^* genotype cannot cause the abortion of any gamete. In this study, we pyramided the *S^n^* allele of SSSLs to develop WCILs, which carry *S^n^* allele in *indica* HJX74 genetic background. The result was that WCILs showed wide compatibility, which was compatible with both *indica* and *japonica* rice varieties (Figures 4, 5). These results showed that the breeding of ICJLs and WCILs is practicable and that the *indica*–*japonica* hybrid sterility could be overcome by using ICJLs and WCILs.

### The Single-Segment Substitution Line Library Is a Powerful Platform for Developing Wide-Compatible *indica* Lines

The development of WCILs requires pyramiding *S^n^* alleles of multiple hybrid sterility loci to improve compatibility. The breeding of WCILs is a challenging task because it is a time-consuming and laborious technique. First, the *S^n^* alleles of the *S5*, *Sa*, *Sb*, *Sc*, *Sd*, and *Se* loci need to be identified and selected from a wide range of genetic resources. Second, *S^n^* alleles of multiple loci need to be pyramided in an *indica* genetic background by MAS. In addition, WCILs need to have improved traits to be used as parents of *indica–japonica* hybrid rice. Over the past two decades, we have constructed a HJX74-SSSL library, which is used as a platform for rice design (Zhang, 2021). Using this platform, a series of CMS, maintainer, and restorer lines were developed (Dai et al., 2015, 2016; Luan et al., 2019). In this study, we identified *S^n^* alleles at the *S5*, *Sb*, *Sc*, *Sd*, and *Se* loci from the HJX74-SSSL library. Since HJX74, the recipient of SSSLs, carried the *S^n^* alleles at *Sb*, *Sd*, and *Se* loci, but the *S^i^* alleles at *S5*, *Sa*, and *Sc* loci, the SSSLs carrying *S5^n^* or *Sc^n^* alleles were selected from the HJX74-SSSL library (Figures 2, 3). The *Sc^n^* and *S5^n^* of the SSSLs were then pyramided in the HJX74 genetic background. Nine WCILs carrying *S^n^* alleles at the *S5*, *Sb*, *Sc*, *Sd*, and *Se* loci in the HJX74 genetic background were developed (Figures 4, 5). The results show that the HJX74-SSSL library is a powerful platform for developing WCILs possessing the complex trait of wide compatibility.

### Wide-Compatible *indica* Lines Will Be Used to Develop *indica–japonica* Hybrid Rice

It is believed that inter-subspecific hybrids have stronger heterosis than intra-subspecific hybrids (Fu et al., 2014; Birchler, 2015). Therefore, the exploitation of inter-subspecific heterosis for the production of improved rice varieties has long been considered (Cheng et al., 2007; Zhang, 2020). The main obstacle in utilizing inter-subspecific heterosis in rice is the *indica–japonica* hybrid sterility. In this study, WCILs were developed using the HJX74-SSSL platform. The WCILs had compatibility with a wide range of *japonica* varieties (Figure 5 and Supplementary Table 13). Therefore, the development of WCILs is an effective approach to overcoming the problem of *indica–japonica* hybrid sterility in breeding practice. By further improving their fertility restoration ability, WCILs can be improved to produce wide-compatible *indica* restorer lines (WCIRLs). Using the HJX74-SSSL platform, a series of WCIRLs is being developed and will be used to develop *indica–japonica* hybrid rice by crossing with *japonica* male sterile lines. Therefore, it is expected that *indica–japonica* hybrid rice will be the rice of next generation (Zhang, 2022).

## Data Availability Statement

The original contributions presented in the study are included in the article/Supplementary Material, further inquiries can be directed to the corresponding author/s.

## Author Contributions

GZ designed and supervised the work, analyzed the data and wrote the manuscript. JG, YL, and LX performed most of the experiments and compiled the experimental data. TY, JZ, ZD, GT, KS, XL, WY, and QT conducted a part of the experiments. HZ, RZ, and SW prepared the experimental materials and supervised some experiments. All authors read and approved the final manuscript.

## Conflict of Interest

The authors declare that the research was conducted in the absence of any commercial or financial relationships that could be construed as a potential conflict of interest.

## Publisher’s Note

All claims expressed in this article are solely those of the authors and do not necessarily represent those of their affiliated organizations, or those of the publisher, the editors and the reviewers. Any product that may be evaluated in this article, or claim that may be made by its manufacturer, is not guaranteed or endorsed by the publisher.

## Footnotes

1
https://www.originlab.com
